# Development of graphene oxide-based biosensing platforms for label-free bioelectronic detection of pathogenic microorganisms

**DOI:** 10.55730/1300-0527.3693

**Published:** 2024-07-17

**Authors:** Sezin YÜKSEL, Fethiye Ferda YILMAZ, Hüseyin TAŞLI, Pınar KARA

**Affiliations:** 1 Department of Analytical Chemistry, Faculty of Pharmacy, Ege University, İzmir, Turkiye; 2 Department of Biomedical Technologies, Graduate School of Natural and Applied Sciences, Ege University, İzmir, Turkiye; 3 Department of Pharmaceutical Microbiology, Faculty of Pharmacy, Ege University, İzmir, Turkiye

**Keywords:** Nanogenosensor, graphene oxide, electrochemical impedance spectrometry, pathogenic microorganisms

## Abstract

A novel electrochemical nanogenosensor devoted to clinical analysis is reported for label-free detection of pathogenic microorganisms in the present work. The designed biosensor is composed of graphene oxide-modified disposable pencil graphite electrodes as a sensing platform. *Escherichia coli* is used as a model case. A 5′-aminohexyl-linked 22-base sequence probe representing the *E. coli* amplicon is immobilized onto sensor surfaces via carbodiimide chemistry. Hybridization is performed with denatured PCR amplicons of the bacteria. Detection is realized by transduction with the electrochemical impedance spectrometry technique. The selectivity of the designed genosensor is measured using *Mycobacterium tuberculosis* and *Klebsiella pneumoniae* sequences. Outstanding sensitivity is achieved with this genosensor array platform with a detection limit of 102 nM. This platform offers promise for rapid, simple, and cost-effective detection of various pathogenic microorganisms.

## 1. Introduction

Pathogenic microorganisms are infectious agents that cause various infectious diseases and even death. They include fungi, protozoa, viruses, and bacteria that can cause various infectious diseases in humans, plants, and animals. Despite the victories won with vaccines and antibiotics against infectious diseases, new and multidrug-resistant pathogenic microorganisms are constantly emerging. They are estimated to be responsible for more than 15 million deaths and major economic losses worldwide every year by infecting humans, plants, and animals through air, water, and food [1–4]. *Escherichia coli* is one of the most infectious agents that cause infectious diseases. Many identification techniques have been developed because the early diagnosis of pathogenic microorganisms that cause infectious diseases is very important. These techniques include both conventional and more recent analysis techniques such as cell culturing, enzyme-linked immunosorbent assay (ELISA), gene sequencing, polymerase chain reaction (PCR), and microarray technology. Most conventional techniques are time-consuming and labor-intensive. Therefore, it is of significant importance to develop fast, sensitive, and cost-effective alternative analytical techniques for the diagnosis of pathogens [5–8].

Biosensors, which are a type of diagnostic technology, are important due to various advantages. Genosensors, or DNA biosensors, are commonly employed to detect sequence-specific DNA. DNA sequences are used as the recognition element in genosensors. The hybridization event between the DNA probe sequence and the target DNA sequence is converted into a quantitative signal and analyzed by the converter. Genosensing technologies have found a variety of applications in many studies, including genetic and infectious disease diagnosis, detection of infectious agents, biomedical and drug detection, applications in the food industry, and environmental pollution monitoring [9–14]. Genosensors are achieved using various transducers, including optical, electrochemical, piezoelectric, and thermal transducers [15].

Electrochemical genosensors have advantages such as high sensitivity, selectivity, simplicity of use, capabilities for fast data analysis, miniaturized structures, compatibility with point-of-care (POC) analysis, and being inexpensive and less time-consuming. Despite these advantages, there are still some issues to be considered, such as low sensitivity in some cases, low stability, and susceptibility to sample matrix effects in analyte detection [16–18]. Electrochemical impedance spectroscopy (EIS) has a significant role in the electrochemical measurements of molecular interactions and various biosensing applications [19,20]. EIS is a powerful method that detects different interface and solution properties of an electrode and is used to detect differences occurring on electrode surfaces. In EIS applications, the Randles circuit, involving charge transfer resistance (Rct), double-layer capacitance (Cdl), solution resistance (Rs), and diffusion processes (Zw), is used to characterize the properties of the electrode/electrolyte interface. EIS measurement values are generally plotted using Nyquist plots. Nyquist plot Im(Z) is plotted against Re(Z) in the frequency range examined. Rct at the electrode surface is calculated using the semicircle diameter that increases when the analyte binds with the target molecule [21,22]. The EIS method is based on the measurement of changes in Rct caused by interactions between the target and the bioreceptor. In genosensor applications, detection of the hybridization of the immobilized DNA probe and complementary DNA target on the sensor surface is performed with this method [23,24].

Biosensors offer significant opportunities for advances in nanotechnology and nanomaterials with diameters ranging between 1 and 100 nm [25,26]. Nanomaterials have small size, high electrical and thermal conductivity, good electron transfer kinetics, biocompatibility, and large surface area. These features allow biosensors to detect bioreceptors with better selectivity and sensitivity and improve their overall performance [27,28]. Graphene derivatives such as graphene oxide (GO) and reduced graphene oxide (rGO) are the preferred carbon-based nanomaterials for biosensor manufacturing, mainly due to their biocompatibility properties. rGO has high electrical conductivity [29,30], while GO has significant characteristics such as the inclusion of oxygenated functional groups, a hydrophilic structure, good stability, low cost, and high catalytic ability [31,32]. Graphene and its derivatives are used in various applications thanks to superior properties like large surface area, good biocompatibility, high mechanical and chemical stability, low toxicity, fast heterogeneous electron transfer, and flexibility [33,34].

In this study, we present a novel approach to electrochemical genosensing for the detection of pathogenic microorganisms by using GO-modified pencil graphite electrodes (GO-PGEs). *E. coli* oligonucleotides and PCR amplicons were used as a model case. The hybridization of complementary and noncomplementary sequences with a DNA probe immobilized to the GO-PGE was monitored using EIS in phosphate-buffered saline (PBS) containing 5 mM [Fe(CN)_6_]^3−/4−^. The surface characterization of PGEs and GO-PGEs was performed by scanning electron microscopy (SEM). The analytical parameters were optimized. The optimized nanogenosensor successfully detected real PCR samples. The designed genosensor enables reliable, fast, cost-effective, POC-compatible, label-free detection and can be applied simply for other pathogenic microorganisms.

## 2. Materials and methods

### 2.1. Apparatus

EIS measurements were performed using the μ-AUTOLAB electrochemical analysis system (Eco Chemie, Utrecht, the Netherlands) with the NOVA 1.10 software package. The three-electrode system consisted of a 3-cm graphite rod and disposable PGEs of 0.5 mm in diameter as the working electrode (WE), a platinum wire as the auxiliary electrode (CE), and an Ag/AgCl reference electrode (RE).

Morphological characterization for the surfaces of PGEs and GO-PGEs was performed by SEM (Model 300VP, Carl Zeiss, Oberkochen, Germany). Fourier transform infrared (FTIR) spectrum analysis was performed with a PerkinElmer Spectrum 100 device (PerkinElmer, Waltham, MA, USA).

### 2.2. Chemicals

Graphene oxide (GO) was obtained from Nanografi (Ankara, Turkey). [N-(3-Dimethylamino)propyl)]-N’-ethylcarbodiimide (EDC), N-hydroxysulfosuccinimide sodium salt (NHS), and Trizma hydrochloride were procured from Sigma-Aldrich Chemical Company (Taufkirchen, Germany) and trisodium citrate dihydrate and sodium dodecyl sulfate (SDS) were procured from Merck (Darmstadt, Germany). Other chemicals used were of analytical reagent grade and obtained from Merck or Sigma.

The 22-mer synthetic oligonucleotide of the *E. coli* specific probe and *E. coli* target sequences were obtained as lyophilized powders from Ella Biotech (Fürstenfeldbruck, Germany). The 81-mer synthetic oligonucleotide of the *Mycobacterium tuberculosis* target sequences was obtained as lyophilized powder from TIB MOLBIOL (Berlin, Germany). *E. coli* PCR amplicons and noncomplementary *K. pneumoniae* amplicons were obtained from the Ege University Faculty of Pharmacy’s Department of Pharmaceutical Microbiology (İzmir, Türkiye). The nucleic acid base sequences of all oligonucleotides and amplicons are shown in Table 1.

All oligonucleotide stock solutions (1000 μg/mL) were prepared with ultrapure water (18 MΩ, Millipore, Darmstadt, Germany), and the oligonucleotides and PCR amplicons were stored at −20 °C.

The various dilutions of *E. coli* probes were prepared in 0.5 M acetate buffer solution (ABS; 0.5 M acetic acid, 20 mM NaCl, pH 4.8), and synthetic target sequences and PCR amplicons were diluted with hybridization buffer solution (HB; 5X SSC; 0.075 M sodium citrate dihydrate, 0.75 M NaCl, pH 7.0). After hybridization, washing was carried out with washing buffer (1X SSC and 0.2% SDS) to remove unbound sequences.

### 2.3. Methods

#### 2.3.1. PCR amplification from *E. coli* isolates

PCR was conducted in a reaction mixture of 50 μL containing 50 ng of DNA, 1X PCR buffer, 1.5 mM MgCl_2_, 1 μM primer, 0.2 mM dNTPs, and 1.25 U of Taq DNA polymerase (Thermo Scientific, Altrincham, UK). The PCR device (Techne, Barloworld Scientific Ltd., London, UK) was programmed for amplification as follows: 1 cycle at 95 °C for 5 min; 30 cycles at 94 °C (50 s), 50 °C (40 s), and 72 °C (1 min); and a final extension step at 72 °C for 10 min. The PCR products were visualized with a UV light screening system (Fusion-FX7, Vilber Lourmat, Collégien, France) after electrophoresis on 1% agarose gel at 100 V for 30 min in TBE buffer [35].

#### 2.3.2. Quantitative determination of samples by spectrophotometric assay

A UV-Vis spectrophotometer (Shimadzu, Tokyo, Japan) was used with quartz cuvettes with volume of 1 mL and 10-mm path length (Starna, Ilford, UK). The concentrations of all oligonucleotides and PCR amplicons were determined by following this method; the A260 unit of double-stranded DNA represented 50 μg/mL, while single-stranded DNA represented 33 μg/mL.

#### 2.3.3. Fabrication of graphene nanomodified sensor surfaces

The interactions between electrochemically reduced graphene oxide (rGO) and GO-nanomodified sensor surfaces and fish sperm dsDNA were investigated. The studies continued with the GO method.

##### 2.3.3.1. Fabrication of electrochemically reduced graphene oxide (rGO) sensor surfaces and dsDNA interaction

GO (1000 μg/mL, 1:1) was prepared in ABS (pH 4.8) and sonicated for 2 h. First, PGEs were activated in 0.5 M ABS (pH 4.8) by applying 1.4 V for 60 s. The prepared GO solution was coated with rGO on the PGE surface by cyclic voltammetry technique. Cyclic voltammetry took place in a potential range of −1.1 V to 1.3 V, with seven cycles and a scan rate of 50 mV s^−1^ [36]. rGO-PGEs were chemically activated for 1 h in PBS (pH 7.4) containing 5 mM EDC and 8 mM NHS. Next, the rGO-PGEs were immobilized in ABS (pH 4.8) containing 10 μg/mL fish sperm dsDNA for 1 h. EIS measurements were monitored (see Figure S1).

##### 2.3.3.2. Fabrication of GO sensor surfaces and dsDNA interaction

GO (400 μg/mL) was prepared in dimethyl sulfoxide (DMSO) and sonicated for 1 h. First, PGE electrodes were pretreated electrically by applying 1.4 V for 60 s in ABS (pH 4.8) and then they were accumulated with PBS (pH 7.4) containing 5 mM EDC and 5 mM NHS to activate the carboxyl groups on the surfaces for 1 h. PGEs were modified in GO solution for 15 min and GO-PGEs were dried for 5 min [37]. GO-PGEs were immobilized in ABS (pH 4.8) containing 10 μg/mL fish sperm dsDNA for 1 h. EIS measurements were monitored (Figure S2).

#### 2.3.4. Electrochemical assay

In this study, an impedimetric genosensor enriched with GO nanomaterial was developed for the detection of pathogenic microorganisms. *E. coli* was used as a model microorganism. Hybridization process steps were carried out on GO sensor surfaces and monitored by EIS transduction.

**Figure 1:**
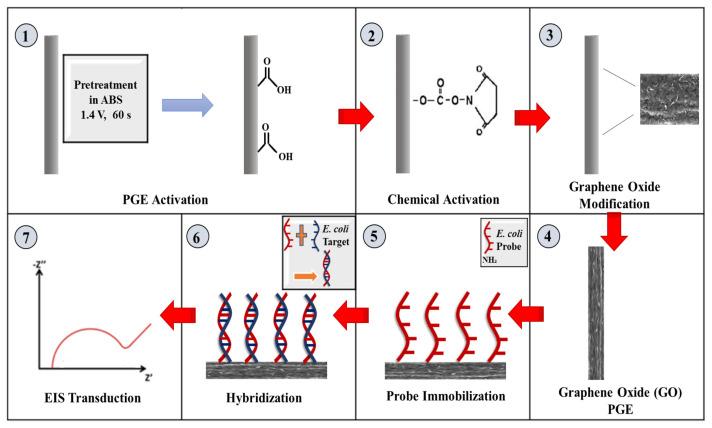
Electrochemical schematic presentation of the nanogenosensor developed for the diagnosis of pathogenic microorganisms.

The experimental procedure steps, as demonstrated in Figure 1, included functionalization of the PGEs and fabrication of the nanogenosensor, hybridization, and impedimetric transduction.

##### 2.3.4.1. Functionalization of the PGEs and fabrication of the nanogenosensor

**Figure 2:**
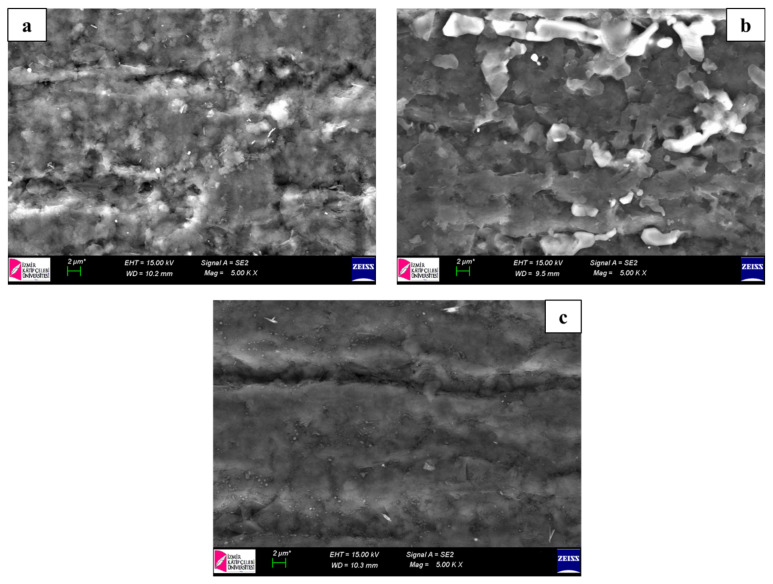
SEM images of bare PGE (a), chemical activation-modified PGE (b), and GO-modified PGE (c) at an acceleration voltage of 15 kV with resolution of 2 μm.

PGE electrodes were pretreated electrically by applying 1.4 V for 60 s in ABS (pH 4.8), and then they were accumulated with PBS (pH 7.4) containing 5 mM EDC and 5 mM NHS to activate the carboxyl groups on the surfaces for 1 h. First, 400 μg/mL GO (3 nm in diameter) was sonicated in DMSO for 1 h at room temperature. Chemically activated surfaces were then incubated with GO solution for 15 min to obtain GO-nanomodified surfaces and dried for 5 min [37]. The GO-PGE surfaces were characterized using SEM as shown in Figure 2. After functionalization of the electrodes, the 5′-aminohexyl linked *E. coli* probe sequence was immobilized onto GO-PGE sensing platforms for 1 h.

##### 2.3.4.2. Hybridization

Both synthetic oligonucleotides and denatured PCR amplicons were hybridized with *E. coli* probe sequences by placing the electrodes into hybridization buffer solutions for 30 min. A boiling water bath was used to thermally denature double-stranded PCR amplicons (8 min at 95 °C); amplicon strand reannealing was blocked by cooling the sample in an ice-water bath for 2 min. Both the PCR blank and noncomplementary PCR amplicons were utilized as negative controls.

##### 2.3.4.3. Impedimetric transduction

EIS was used to detect hybridization. EIS measurements were performed in PBS containing 5 mM Fe[CN_6_]^3−/4−^ by applying 0.24 V. An impedance frequency range from 10 kHz to 50 MHz and an AC amplitude of 10 mV were applied. Nyquist diagrams were analyzed using the Randles equivalent circuit. In the Nyquist diagram, charge transfer resistance (Rct) is related to the semicircle diameter. The Randles equivalent circuit was used for fitting to the impedance data.

All results presented in this study are the means of at least five measurements and error bars correspond to standard deviations.

## 3. Results and discussion

In this study, a label-free impedimetric nanogenosensor was improved for the direct detection of pathogenic microorganisms. PGE surfaces were nanomodified with GO nanomaterial to achieve a larger surface area and higher conductivity. An *E. coli* probe sequence was immobilized onto GO-PGEs via carbodiimide chemistry. Detection was performed by EIS transduction and monitoring of the hybridization between the probe and the target sequences on the nanomodified sensor surfaces.

### 3.1. Morphological characterization of the electrodes

The surface morphology of the electrodes was determined for each parameter using SEM (Model 300VP, Carl Zeiss, Oberkochen, Germany). The morphological characterization of the bare PGE, chemical activation-modified PGE, and 3-nm-diameter GO-modified PGE was performed using SEM at an acceleration voltage of 15 kV with resolution of 2 μm. Figure 2 shows SEM images of bare PGE, chemical activation-modified PGE, and GO-modified PGE. Figure 2a displays the unmodified PGE surface with a very rough structure. A brighter surface and film layer were observed on the PGE surface obtained after chemical activation (Figure 2b) compared to the bare PGE (Figure 2a). After GO modification on the PGE surface (Figure 2c), changes and more layers were observed compared to the chemical activation-modified PGE (Figure 2b). When the SEM images were examined, it was concluded that GO modification occurred on the PGE surface, similar to the results reported in recent studies [38].

FTIR spectrometry (PerkinElmer Spectrum 100 device, PerkinElmer, Waltham, MA, USA) was performed to investigate the molecular interactions of GO. The FTIR spectra gave the characteristic vibration bands of GO, including carbonyl groups, aromatic rings, and epoxy groups (Figure S3).

The surface areas of bare and GO-modified PGEs were evaluated using cyclic voltammetry by scanning from −0.5 to 0.5 in 1 mM [Fe(CN)_6_]^3−/4−^. The surface areas of the electrodes were calculated with the Randles–Sevcik equation. According to that equation, the surface area of the bare PGE was 0.24 cm^2^, while the surface area of the GO-PGE was calculated as 0.34 cm^2^.


$$\text{Ip}=2.69\times 10^{5}\times {\text{n}}^{3/2}\;{\text{AD}}^{1/2}\;{\text{v}}^{1/2}\;\text{C}$$

In the equation above, Ip = peak current (in A), n = number of electrons participating in the redox reaction, A = electrode surface area (in cm^2^), C = concentration of the redox probe (in mol cm^−3^), D = diffusion coefficient (in cm^2^ s^−1^), and V = scan rate (in V s^−1^) [39].

### 3.2. Electrochemical characterization

**Figure 3:**
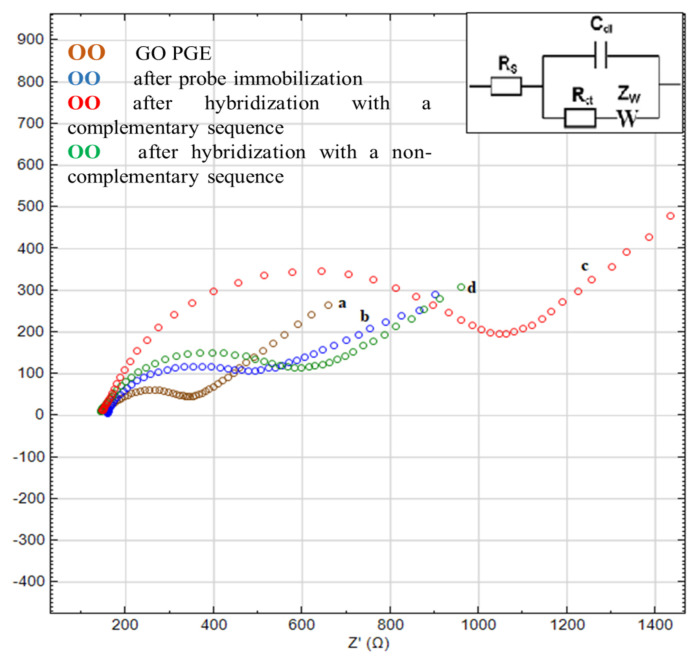
Nyquist plot of the initial GO-PGE (a), after probe immobilization (b), after hybridization with a complementary sequence (c), and after hybridization with noncomplementary sequence (d) for electrodes containing 5 mM [Fe(CN)_6_]^3−/4−^ in PBS solution (pH 7.4). Inset: Randles circuit.

Electrochemical characterization of the designed nanogenosensor was performed by monitoring the EIS transduction of hybridization between synthetic sequences. The respective curves in Figure 3 show the Nyquist plots obtained before *E. coli* probe immobilization, before hybridization, and after hybridization with the *E. coli* target sequence and the noncomplementary (NC) *M. tuberculosis* sequence at the GO-PGE surface in the presence of 5 mM [Fe(CN)_6_]^3−/4−^.

According to the electrochemical characterization results, the Rct value of the GO-PGE decreased when GO was modified on the bare PGE surface (bare PGE data not shown). This decrease may be attributed to increased conductivity at the sensor surface and higher electron transfer between the electrode and electroactive species in the solution [40]. The Rct value of the DNA probe immobilized on the GO sensor surface increased (Figure 3, curve b). This increase was due to the electrostatic pull force between the negative phosphate groups on the DNA probe surface and the [Fe(CN)_6_]^3−/4−^ redox probe [41]. The Rct values increased even more with hybridization between the DNA probe and the target sequence (Figure 3, curve c). This increase prevented the driving force of the negatively charged [Fe(CN)_6_]^3−/4−^ redox probe due to the backlog of more negative charges from the DNA probe and complementary DNA [42]. Therefore, Rct values increased due to the formation of double-stranded DNA resulting from the interaction between the probe and target DNA. A slightly increased Rct value was obtained by hybridization between the DNA probe and the noncomplementary sequence used for the selectivity of the nanogenosensor (Figure 3, curve d). This hybridization confirms the selectivity of the nanogenosensor. These results show that the nanogenosensor was achieved successfully.

ΔRct% values were calculated for each optimum hybridization condition according to the relevant equation of [(Rct_probe+target_ – Rct_probe_) / Rct_probe+target_] × 100% and the results are shown in Table 2.

Looking at the values from this calculation, Rct_probe_ is the resistance obtained after immobilizing the *E. coli* probe sequence on the GO sensor surfaces. The Rct_probe_ + FM_target_ and Rct_probe_ + NC_target_ (*E. coli* and *M. tuberculosis* sequences) values are the resistance obtained after interaction between the probe and target sequences. The differences between these specified values provide information about the best degree of bonding of the nanogenosensor.

### 3.3. Analytical characterization

In a series of studies conducted using the synthetic *E. coli* probe and target sequences, optimal hybridization conditions were determined to enhance the selectivity and sensitivity of the nanogenosensor. The selectivity of the nanogenosensor was determined by evaluating the difference between Rct values acquired after hybridization with the complementary and NC targets (*M. tuberculosis* sequences) as analytical responses in the form of FM/NC (FM: full match; NC: noncomplementary). The results of the optimization studies, including the parameters used and the results obtained, are shown in Table 3.

Optimal hybridization and selectivity conditions were determined by considering high FM/NC ratios as analytical responses.

**Figure 4:**
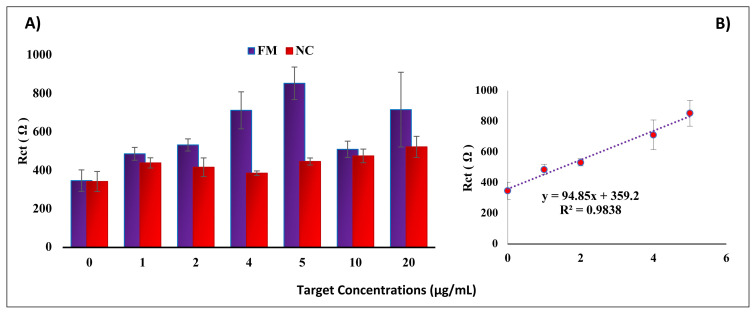
A) Histogram showing Rct values acquired for different target concentrations on steady probe-coated GO sensor surfaces after hybridization, ranging from 0 to 20 μg/mL target concentrations. B) Calibration plot for Rct values in the presence of different target sequence concentrations, ranging from 0 to 5 μg/mL.

First, Rct values were investigated by applying probe concentrations ranging from 0.1 to 10 μg/mL on GO sensor surfaces. It was determined that the selectivity and FM/NC ratios increased up to 1 μg/mL, with the highest FM/NC ratio observed at 1 μg/mL. The probe concentration was then kept fixed at 1 μg/mL with application of increasing target concentrations ranging from 0 and 20 μg/mL. The Rct values reached the selected target concentration of 5 μg/mL and then decreased (Figure 4A). Furthermore, a high FM/NC ratio was obtained at a target concentration of 5 μg/mL. After determining the optimum probe concentration of 1 μg/mL and target concentration of 5 μg/mL, other analytical parameters were examined. First, the hybridization and washing buffer parameters were investigated, respectively. The hybridization buffer containing 5X SSC and washing buffer containing 1X SSC + 0.2 SDS were selected considering high selectivity, high sensitivity, and small error bars. Next, probe immobilization time, washing time, and GO modification time were investigated. For these parameters, hybridization selectivity and FM/NC ratio were increased until the selected time and then decreased after that chosen time. The optimal hybridization conditions for these parameters were determined as 60 min, 2 min, and 15 min, respectively (Figure S4). Figure 4B shows the calibration graph for Rct values obtained in the presence of target sequences of different concentrations between 0 and 5 μg/mL on probe-coated GO sensor surfaces to determine the limit of detection. The limit of detection estimated from S/N = 3 reached 102 nM (0.68 μg/mL).

Next, the effect of the nanomaterial on hybridization was investigated. Studies were conducted on GO-modified and non-GO-modified surfaces to examine the effect of nanomaterial modification. For this purpose, interactions occurring on bare PGE and GO-PGE surfaces under the optimum conditions were investigated using the DNA probe sequence and complementary (*E. coli*) and noncomplementary (*M. tuberculosis*) synthetic sequences. As a result, it was determined that higher FM Rct values and better FM/NC discrimination were obtained on PGE surfaces modified with GO compared to bare PGE surfaces (Figure S5). With the developed nanogenosensor, better results were observed. According to these results, the effect of the nanomaterial on the sensitivity and selectivity of the genosensor was determined.

The genosensor presented in this study was compared with other methods in terms of detection performance. That comparison is shown in Table 4.

### 3.4. Quantitative analysis of real samples by agarose gel electrophoresis

**Figure 5:**
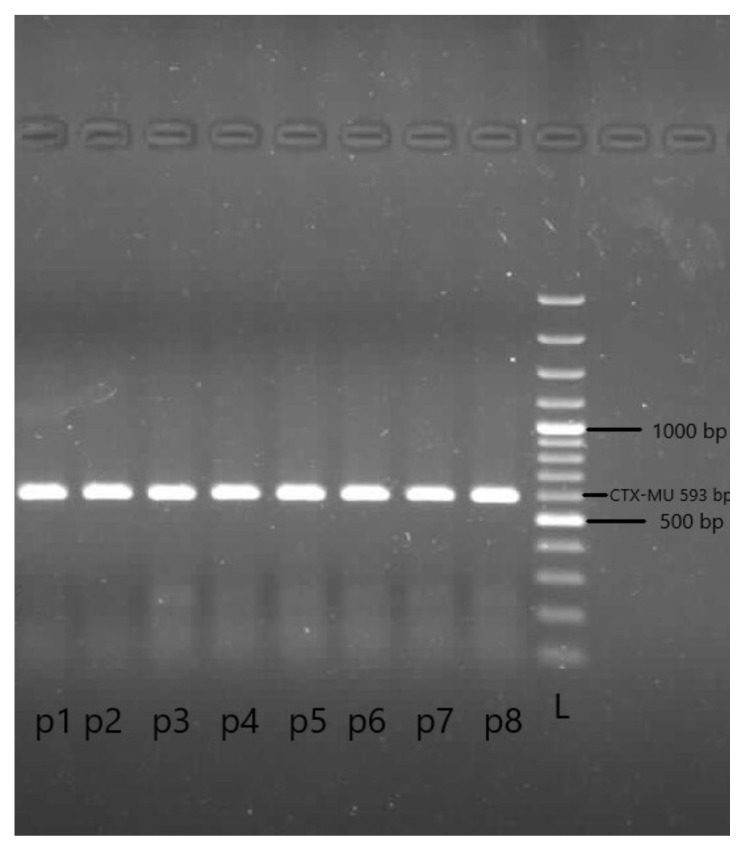
PCR imaging results for the presence of CTX-M. L: 100-bp DNA ladder, p: CTX-MU-positive isolate.

Views of CTX-MU-positive and CTX-MU-negative samples by agarose gel electrophoresis are shown in Figure 5. Ladder and band sizes were compared. As a result of these images and comparisons, the presence of the CTX-M enzyme was proven.

### 3.5. Nanogenosensor application with real samples

**Figure 6:**
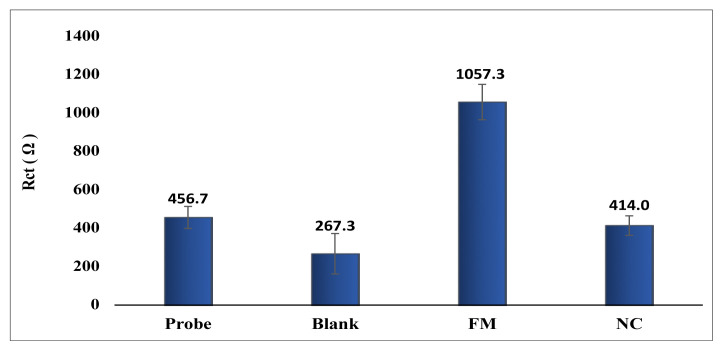
Rct values obtained before and after hybridization with *E. coli* (FM) and NC amplicons and PCR blank solutions on GO-PGE surfaces.

The detection capability of the genosensor with real samples was evaluated under optimum conditions. The Rct values obtained after hybridization with complementary (*E. coli*) amplicons, PCR blanks, and noncomplementary (*K. pneumoniae*) amplicons on GO-PGEs were evaluated. The results are shown in Figure 6. After hybridization with the probe and complementary amplicon (FM), a high Rct value was achieved due to completion properties. After hybridization with the probe and noncomplementary amplicon (NC), a low Rct value was obtained due to lower completion properties. After hybridization with the PCR blank, a low Rct value was obtained, and it was determined that it did not affect the surface.

**Figure 7:**
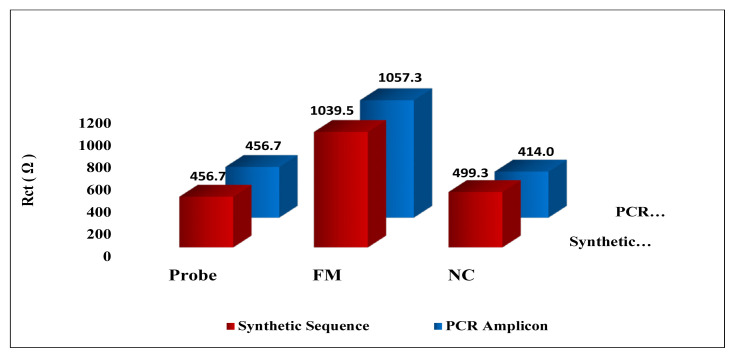
EIS transduction for Rct values obtained for synthetic sequences and denatured PCR amplicons with optimum conditions: probe (before hybridization), FM (after hybridization with probe and complementary target), and NC (after hybridization with probe and noncomplementary target).

Figure 7 shows the Rct values achieved after hybridization with the probe-coated GO sensor surface with synthetic sequences and PCR amplicons. According to the results, while standard Rct values were obtained on probe-coated GO surfaces before hybridization, higher Rct values were obtained after hybridization with the complementary sequence and the complementary PCR amplicon. This result confirmed that hybridization had taken place. With the noncomplementary sequence and noncomplementary amplicon used for evaluating the selectivity of the nanogenosensor, low Rct values were obtained after hybridization. This proved the selectivity of the nanogenosensor. As a result of studies conducted with synthetic sequences and PCR amplicons, it was concluded that the developed nanogenosensor is highly sensitive and specific.

Finally, experiments were performed on probe-free sensor surfaces to verify the selectivity and specificity of the probe DNA sequence compared to the target sequence. In these studies, the interactions of complementary (*E. coli*) and noncomplementary (*M. tuberculosis*) synthetic sequences and complementary PCR amplicons (*E. coli*) and noncomplementary PCR amplicons (*K. pneumoniae*) were investigated using probe-free GO-modified PGE surfaces. As a result, Rct values close to the value of the probe sequence were obtained on probe-free surfaces and no difference was observed (Figure S6). However, high Rct values were obtained after the interaction of the probe sequence with the target sequence and PCR amplicon. These results showed that the *E. coli* probe sequence was specific for the *E. coli* target sequence and the *E. coli* PCR amplicon.

A series of five iterative measurements of synthetic and PCR amplicons on modified probe surfaces before and after hybridization with the complementary and NC amplicons were achieved with relative standard deviations of 8.2%, 10.1%, 9.8%, 8.9%, 9.2%, and 9.7%, respectively.

Table 5 presents the detection results for label-free pathogenic microorganisms by various electrochemical methods. Considering the previous studies, the developed genosensor is comparable with the results presented in Table 5 when selectivity, sensitivity, and ease of application are considered. In addition, our prototype has applicability to other pathogenic microorganisms by using a long nonspecific sequence (*K. pneumoniae*). Furthermore, the designed prototype is applicable to POC systems due to its rapidity, ease of use, and cost-effectiveness.

## 4. Conclusions

A selective and sensitive label-free GO-enriched impedimetric genosensor has been designed for the diagnosis of pathogenic microorganisms in this study. The production of GO sensor surfaces was verified by EIS and SEM. Analytical parameters including probe concentration, target concentration, and hybridization buffer were optimized to determine optimal hybridization conditions and increase the selectivity and sensitivity of the designed nanogenosensor. The selectivity of the nanogenosensor was evaluated by comparing Rct values between target and noncomplementary sequences on the GO sensor surface. After determining the most suitable conditions, the results obtained with PCR samples were evaluated. In comparative studies involving both synthetic sequences and PCR products, it was determined that the designed genosensor was highly selective and sensitive. These results show that a sensitive, selective, cheap, simple, and sturdy electrochemical nanogenosensor was designed for the diagnosis of pathogenic microorganisms. This constitutes a new approach for microchip technologies for improving the selectivity and sensitivity of nanogenosensors for the diagnosis of pathogenic microorganisms or microbiological diseases. This platform offers a fast, simple, POC-compatible, and sensitive approach to the diagnosis and evaluation of other pathogenic microorganisms. With the determined results, this study can help practitioners avoid real-time PCR, which is costly, in contrast to other studies.

## Supplementary Information

### S1

To check whether our nanomaterial can work or not, a range of preliminary experiments were carried out. For this purpose first, the electrochemical behaviors of dsDNA covalently bonded to bare PGEs and rGO modified PGEs were investigated. Figure S1 shows that the histogram plots provided after EIS measurements. The impedance measurements obtained in the presence of bare PGEs and rGO PGEs and dsDNA immobilized on surfaces are shown. A lower Rct value was obtained in rGO PGEs compared to bare PGEs. Because the conductivity and electron transfer rate increased, the resistance decreased. And it was determined that the sensor surface was successfully coated with rGO. Looking at dsDNA immobilized on the sensor surfaces, more dsDNA was attached to rGO PGEs. More dsDNA was bound to rGO-PGE due to the increased resistance to charge transfer and the presence of more carboxyl groups on the electrode surface. Also, this increase after the immobilization of dsDNA on the sensor surface is associated with the slowing of the electron transfer rate as a result of the interaction between the redox probe and the dsDNA negative phosphate groups [1,2].

#### Supplementary Data

Figure S1 Histograms show bare PGE and rGO-PGE surfaces and dsDNA binding to these surfaces acquired by Electrochemical Impedance Spectroscopy transduction of Rct values.

#### S2

The second of the studies to check whether our nanomaterial is working is the graphene oxide method. The impedance measurement of dsDNA covalently bonded to bare PGEs and GO modified PGEs was investigated. Figure S2 shows that the histogram plot of Rct values provided after EIS transduction. GO-PGE has a low Rct value due to its conductivity and surface area with properties. And it was determined that the sensor surface was successfully coated with GO. Looking at dsDNA immobilized on the sensor surfaces, more dsDNA was attached to GO PGEs. More dsDNA was bound to GO-PGE due to the increased resistance to charge transfer and the presence of more carboxyl groups on the electrode surface. Also, this increase after the immobilization of dsDNA on the sensor surface is associated with the slowing of the electron transfer rate as a result of the interaction between the redox probe and the dsDNA negative phosphate groups [1,2].

Figure S2 Histograms show of Rct values of acquired using bare PGE and GO-PGE surfaces and dsDNA binding to these surfaces.

Figure S3 FTIR spectra of Graphene Oxide.

Figure S4 Histograms show Rct values acquired in the presence of 5 mM [Fe(CN)_6_]^3−/4−^ in PBS; A) Probe concentrations, B) Hybridization buffer, C) Washing buffer, D) Washing time, E) Probe immobilization time, F) Graphene Oxide (GO) modification time.

Figure S5 Histogram plot of Rct values provided using; bare PGE and GO-modified PGE under the same conditions: probe (before hybridization), FM (after hybridization with probe and complementary target), NC (after hybridization with probe and non-complementary target).

Figure S6 Histogram plot of Rct values provided using; synthetic sequences and PCR amplicons at optimal conditions probe-free surfaces on GO-PGE sensor surfaces.

References1

Akbari HasanjaniHR
ZareiK

Electrochemical sensor for ultrasensitive determination of ceftazidime using hollow platinum nanoparticles/reduced graphene oxide/pencil graphite electrode
Chemical Papers
2018
72
1935
1944
10.1007/s11696-018-0428-4
2

FindikM
BingolH
ErdemA

Electrochemical detection of interaction between daunorubicin and DNA by hybrid nanoflowers modified graphite electrodes
Sensors and Actuators B: Chemical
2021
329
129120
10.1016/j.snb.2020.129120


## Figures and Tables

**Table 1:** DNA sequences used in the study.

Oligonucleotide	Sequence
*Escherichia coli* probe	5′-NH_2_-C_6_-GGC AGC GGT GAC TAT GGC ACC A -3′
*Escherichia coli* synthetic target (complementary)	5′-TGG TGC CAT AGT CAC CGC TGC C-3′
*Escherichia coli* PCR amplicon (complementary)	5′-AAAAGTGAAAGCGAACCGAATCTGTTAAATCAGCGAGTTGAGATCAAAA AATCTGACCTTGTTAACTATAATCCGATTGCGGAAAAGCACGTCAATGGGAC GATGTCACTGGCTGAGCTTAGCGCGGCCGCGCTACAGTACAGCGATAACGTG GCGATGAATAAGCTGATTGCTCACGTTGGCGGCCCGGCTAGCGTCACCGCGT TCGCCCGACAGCTGGGAGACGAAACGTTCCGTCTCGACCGTACCGAGCCGAC GTTAAACACCGCCATTCCGGGCGATCCGCGTGATACCACTTCACCTCGGGCAATGGC GCAAACTCTGCGGAATCTGACGCTGGGTAAAGCATTGGGCGACAGCCAACGGGCGC AGCTGGTGACATGGATGAAAGGCAATACCACCGGTGCAGCGAGCATTCAGGC TGGACTGCCTGCTTCCTGGGTTGTGGGGGATAAAACCGGCAGCGGTGACTATGGCAC CACCAACGATATCGCGGTGATCTGGCCAAAAGATCGTGCGCCGCTGATTCTGGTCAC-3′
*Mycobacterium tuberculosis* target (noncomplementary, NC)	5′-CAGCCAGCTGAGCCAATTCATGGACCAGAACAACCCGCTGTC GGGGTTGA CCCACAAGCGCCGACTGTCGGCGCTGGGGC-3′
*Klebsiella pneumoniae* PCR amplicon (NC amplicon)	5′-ATGTTAAAAGTTATTAGTAGTTTATTGGTCTACATGACCGCGTCTGTCAT GGCTGTAGCTAGTCCGTTAGCCCATTCCGGGGAGCCGAGTGGTGAGTATCC GACAGTCAACGAATTCCGGTCGGAGAGGTCCGGCTTTACCAGATTGCTGAT GGTGTTTGGTCGCATATCGCAACGCAGTCGTTTGATGGCGCGGTCTACCCA TCCAATGGTCTCATTGTCCGTGATGGTGATGAGTTGCTTTTGATTGATACAG CGTGGGGTGCGAAAAACACAGCGGCCCTTCTCGCGGAGATTGAGAAGCAAA TTGGACTTCCCGTAACGCGTGCAGTCTCCACGCACTTTCATGACGACCGCGT CGGCGGCGTTGATGTCCTTCGGAAGGCTGGAGTGGCAACGTACGCATCACC GTCGACACGCCGGCTAGCCGAGGCAGAGGGGAACGAGATTCCCACGCACTC TCTAGAAGGACTCTCATCGAGCGGGGACGCAGTGCGCTTCGGTCCAGTAGA GCTCTTCTATCCCGGTGCTGCGCATTCGACCGACAATCTGGTTGTATACGTCCCGTC AGCGAACGTGCTATACGGTGGTTGTGCCGTTCTTGCGTTGTCACGCACGTCTGC GGGGAACGTGGCCGATGCCGATCTGGCTGAATGGCCCACCTCCGTTGAGC GGATTCAAAAACACTACCCGGAAGCAGAGGTCGTCATTCCCGGGCACGGTCTAC CGGGCGGTCTAGACTTGCTCCAGCACACAGCGAACGTTGTCACAGCACACAAAA ATCGCTCAGTCGCCGAGTAG-3′

**Table 2:** ΔRct% values of the effects of probe concentration, target concentration, hybridization and washing buffers, probe immobilization, and washing and graphene oxide modification times on nanogenosensor. Best values are provided in bold font.

ΔRct%	Probe concentration (μg/mL)							

		0.1	0.2	0.5	1	2	5	10
	
	FM	63	64.1	65.3	**66**	47.9	37.7	37.5
	
	NC	60.3	59.5	58.4	**55**	41.2	32	28.3

ΔRct%	Target concentration (μg/mL)							

		0	1	2	4	5	10	20
	
	FM	48.3	56.7	58.9	64.4	**69.7**	57.9	65
	
	NC	48	54.2	52.9	51	**54.6**	56.2	58.5

ΔRct%	Hybridization buffer							

		0.5X SSC		1X SSC	2X SSC	3X SSC		5X SSC
	
	FM	52.8		55.7	70	70.1		**72.2**
	
	NC	47.1		49.6	56.5	56.4		**58.1**

ΔRct%	Washing Buffer							

		1X SSC + 0.05% SDS		1X SSC + 0.1% SDS	1X SSC + 0.2% SDS	1X SSC + 0.5% SDS		1X SSC + 1% SDS
	
	FM	61.7		70.8	**74.7**	73.8		71.8
	
	NC	57.5		56.2	**57.9**	57.2		63.5

ΔRct%	Probe immobilization time (min)							

		15		30	45	60	75	90
	
	FM	70		71.1	72.8	**74**	69.8	68.9
	
	NC	59.6		61.9	54.7	**55.3**	55.3	68.6

ΔRct%	Washing time (min)							

		0	0.5	1	2	5	10	15
	
	FM	78.3	75.2	74.3	**74.5**	70.1	67.5	66.2
	
	NC	77.2	66.3	66.2	**56.3**	61.2	55.6	57.5

ΔRct%	Graphene oxide (GO) modification time (min)							

		5		10	15	30	60	
	
	FM	64.1		66.6	**74.2**	69	61.8	
	
	NC	57.4		57.1	**56.2**	59.8	55.8	

**Table 3:** Effects of probe concentration, target concentration, hybridization and washing buffers, probe immobilization, and washing and graphene oxide modification times on the nanogenosensor’s selectivity. Best values are provided in bold font.

	Probe concentration (μg/mL)						
	0.1	0.2	0.5	1	2	5	10
FM/NC	1.2	1.3	1.4	**1.6**	1.3	1.3	1.5
	Target concentration (μg/mL)						
	0	1	2	4	5	10	20
FM/NC	1.0	1.1	1.3	1.6	**1.9**	1.1	1.4
	Hybridization buffer						
	0.5X SSC	1X SSC		2X SSC	3X SSC		5X SSC
FM/NC	1.3	1.3		1.8	1.8		**1.9**
	Washing buffer						
	1X SSC + 0.05% SDS	1X SSC + 0.1% SDS		1X SSC + 0.2% SDS	1X SSC + 0.5% SDS		1X SSC + 1% SDS
FM/NC	1.2	1.9		**2.1**	2.1		1.5
	Probe immobilization time (min)						
	15	30	45	60		75	90
FM/NC	1.5	1.4	1.9	**2.1**		1.9	1.0
	Washing time (min)						
	0	0.5	1	2	5	10	15
FM/NC	1.1	1.5	1.5	**2.1**	1.5	1.7	1.4
	Graphene oxide (GO) modification time (min)						
		5	10	15	30	60	
	FM/NC	1.3	1.5	**2.1**	1.5	1.3	

**Table 4:** Comparison of different methods developed for the detection of pathogenic microorganisms.

Detection method	LOD/LOQ	Reference
Electrochemical genosensor (DNA biosensor)	0.68 μg/mL	This work
Surface plasmon resonance (SPR)	11.7 μg/mL	[43]
Quartz crystal microbalance (QCM) immunosensor	145 μg/mL	[44]
Culture assays	16%	[45]
PCR	80%	[46]
Quantitative PCR	10^3^–10^4^ CFU/mL	[47]

%: Percentage of identified cases from among all samples.

**Table 5:** Classification of label-free electrochemical biosensors for detection of pathogenic microorganisms.

Target pathogen	Electrode	Biorecognition element	Nanomaterial interface	LOD/LOQ	Ref.
*E. coli*	Pencil graphite electrode (PGE)	ssDNA probe	Graphene oxide (GO)	0.68 μg/mL	This work
*Salmonella typhimurium*	GCE	Anti-*S. typhimurium*	AuNPs/PAMAM-MWCNT-Chi	5 × 10^2^ CFU/mL	[48]
*Mycobacterium tuberculosis*	Nanotube array electrode	ssDNA probe	Gold	50 ng/mL	[49]
*S. typhimurium*	SPCE	*S. typhimurium* aptamer	AuNPs	6 × 10^2^ CFU/mL	[50]
*Neisseria meningitides*	Pt/Si	ssDNA probe	Zinc oxide	5 ng/μL	[51]
*Pseudomonas aeruginosa*	ITO electrode	Pyocyanin toxin	PANI/AuNPs	500 nM	[52]
*Citrus tristeza* virus	SPCE	Thiolated ssDNA probe	AuNPs	100 nM	[53]
