# Supplementary figures and images for: Development of graphene oxide-based biosensing platforms for label-free bioelectronic detection of pathogenic microorganisms
Source: Turk J Chem. 2024 Jul 17;48(5):733–47. doi: 10.55730/1300-0527.3693 (PMC11539911; doi:10.55730/1300-0527.3693)

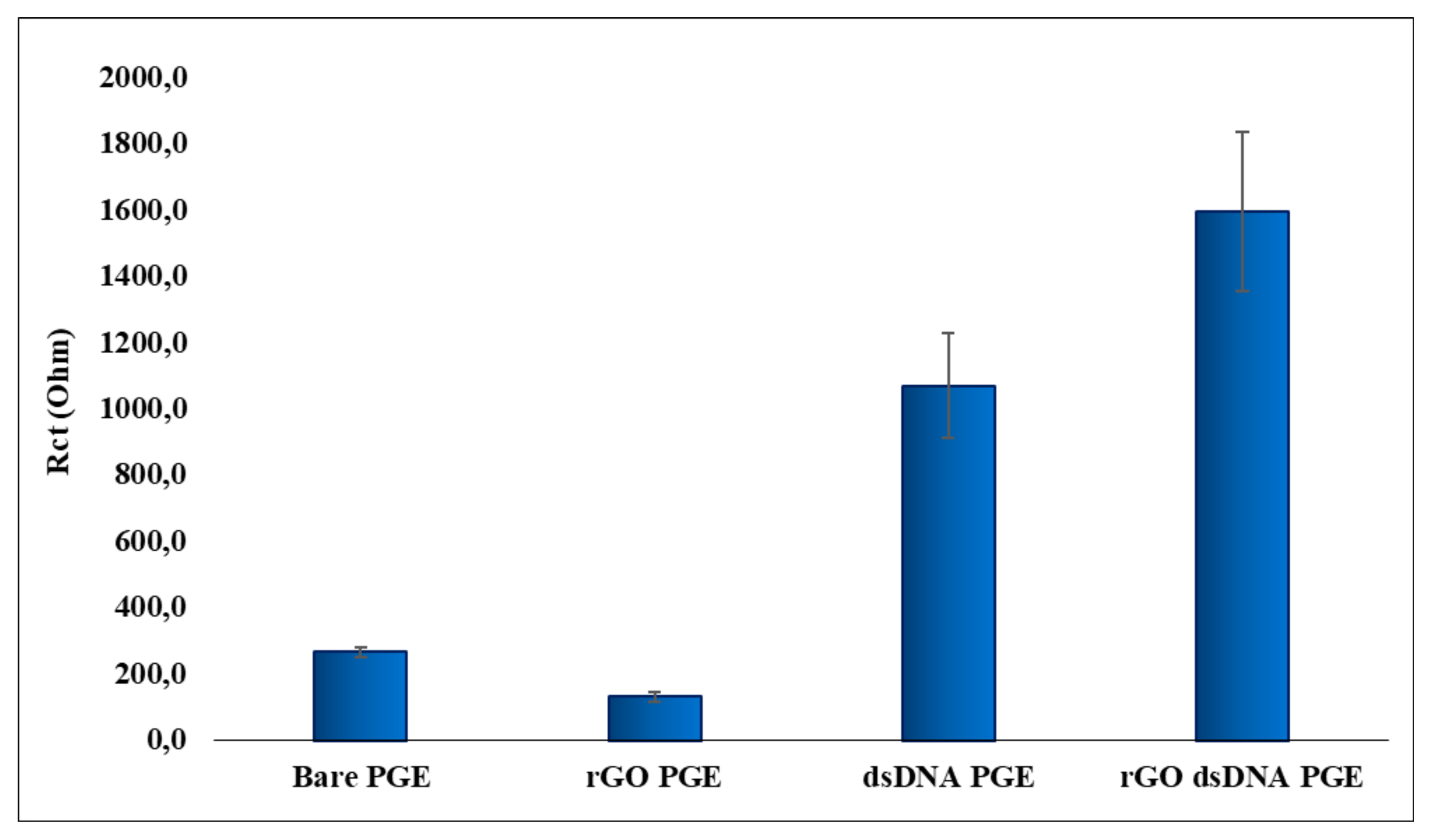

Supplement: Figure S1 — Histograms show bare PGE and rGO-PGE surfaces and dsDNA binding to these surfaces acquired by Electrochemical Impedance Spectroscopy transduction of Rct values. [file tjc-48-05-733s1.tif]

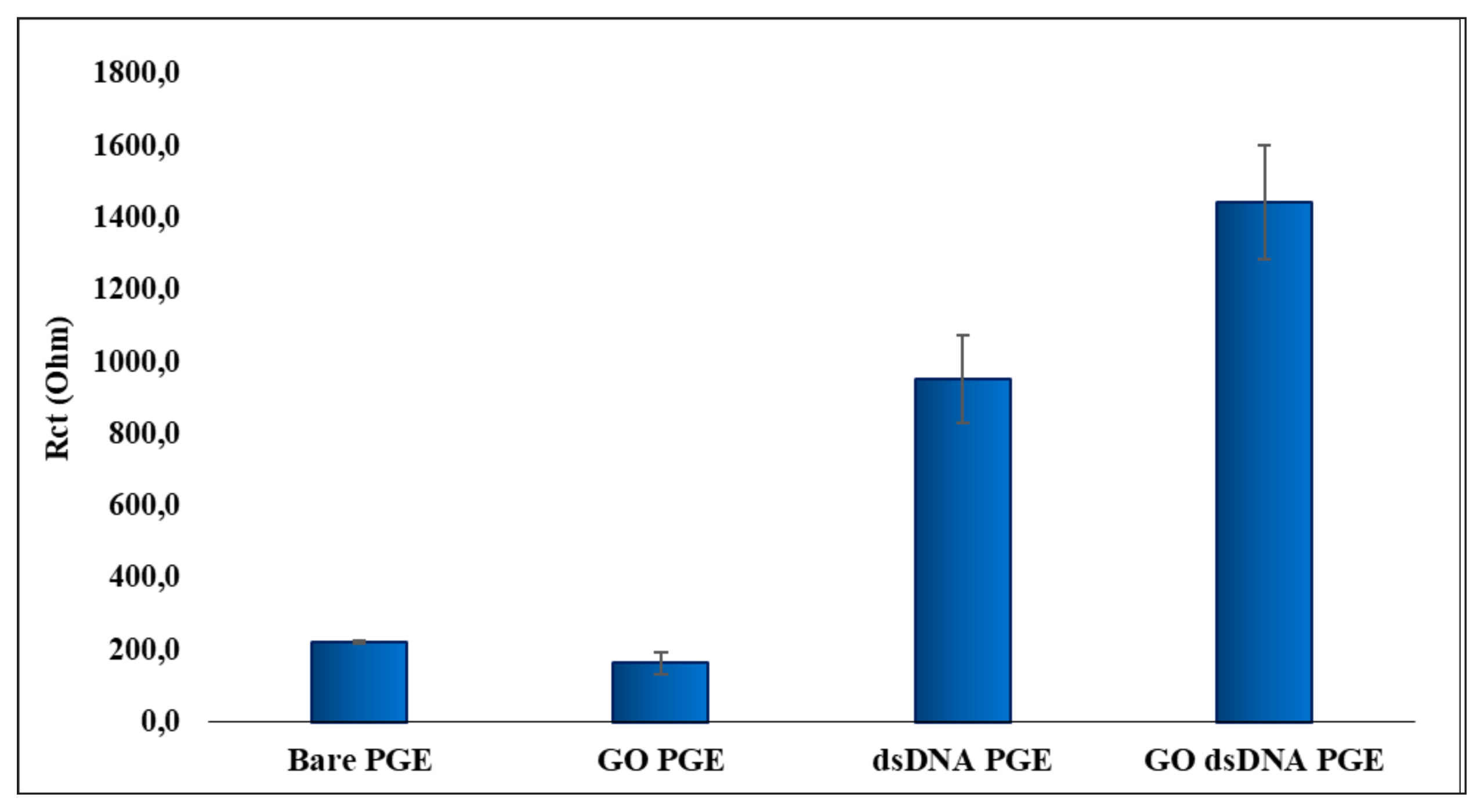

Supplement: Figure S2 — Histograms show of Rct values of acquired using bare PGE and GO-PGE surfaces and dsDNA binding to these surfaces. [file tjc-48-05-733s2.tif]

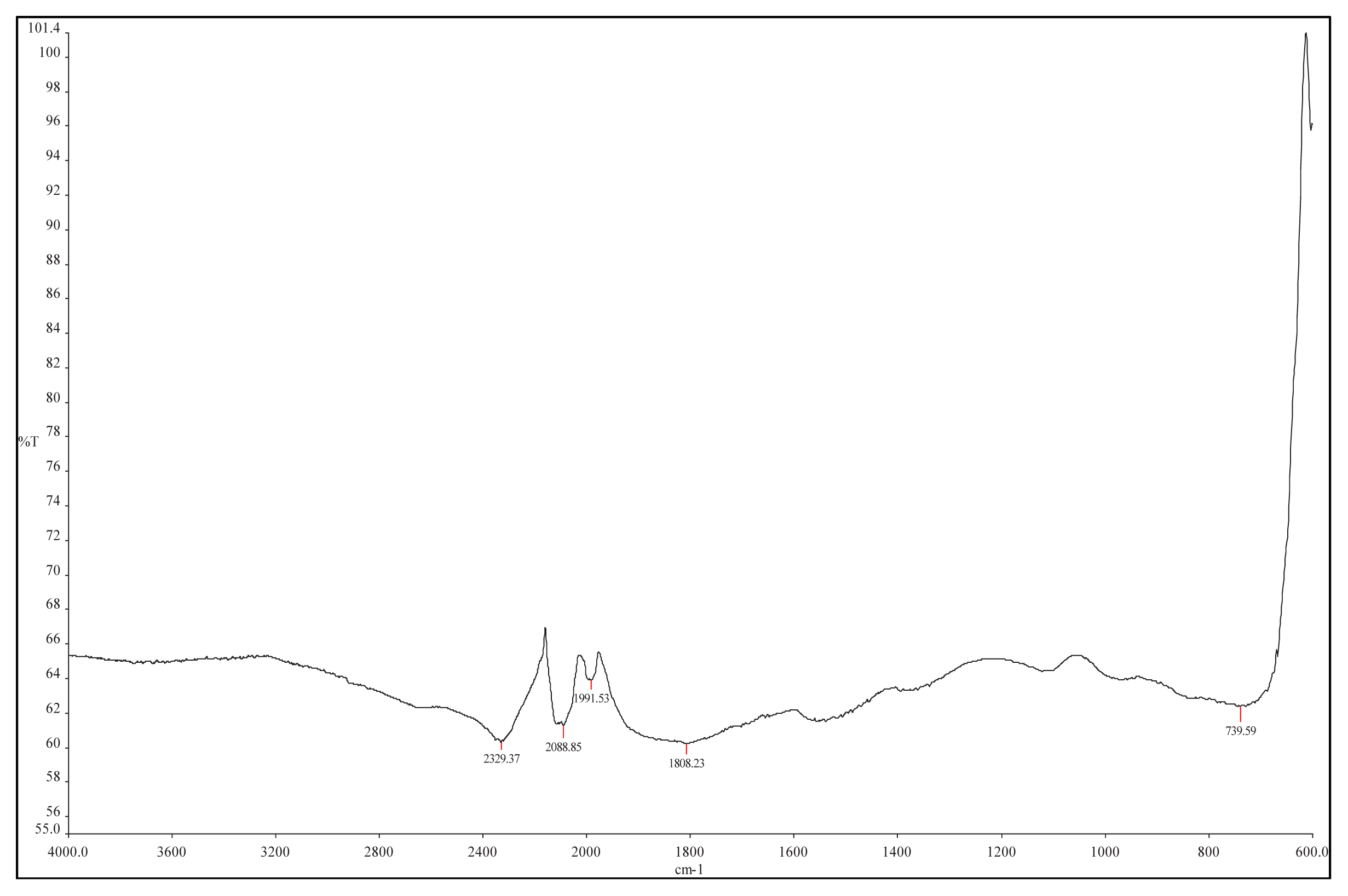

Supplement: Figure S3 — FTIR spectra of Graphene Oxide. [file tjc-48-05-733s3.tif]

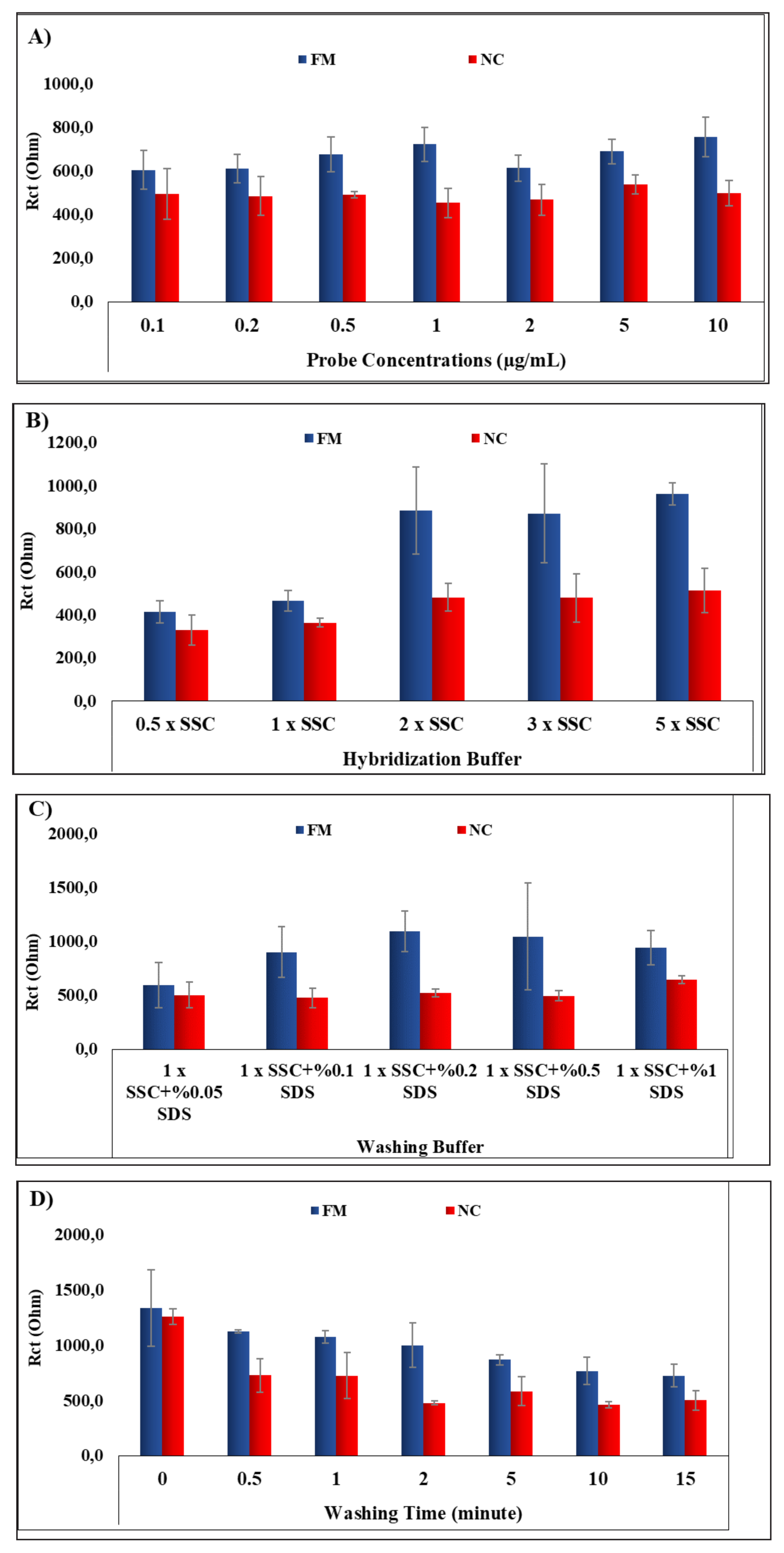

Supplement: Figure S4 — Histograms show Rct values acquired in the presence of 5 mM [Fe(CN)6]3−/4− in PBS; A) Probe concentrations, B) Hybridization buffer, C) Washing buffer, D) Washing time, E) Probe immobilization time, F) Graphene Oxide (GO) modification time. [file tjc-48-05-733s4a.tif]

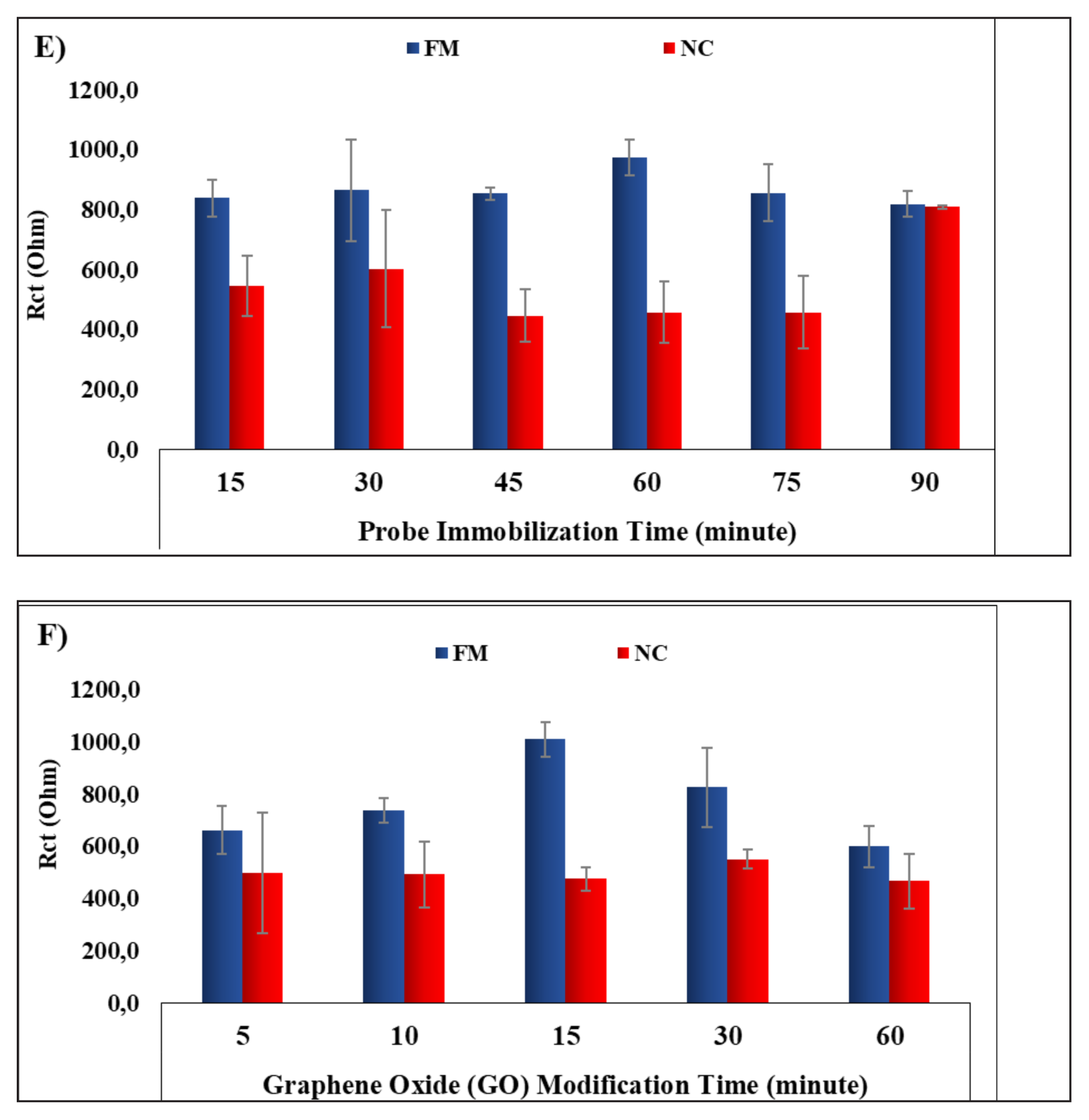

Supplement: Figure S4 — Histograms show Rct values acquired in the presence of 5 mM [Fe(CN)6]3−/4− in PBS; A) Probe concentrations, B) Hybridization buffer, C) Washing buffer, D) Washing time, E) Probe immobilization time, F) Graphene Oxide (GO) modification time. [file tjc-48-05-733s4b.tif]

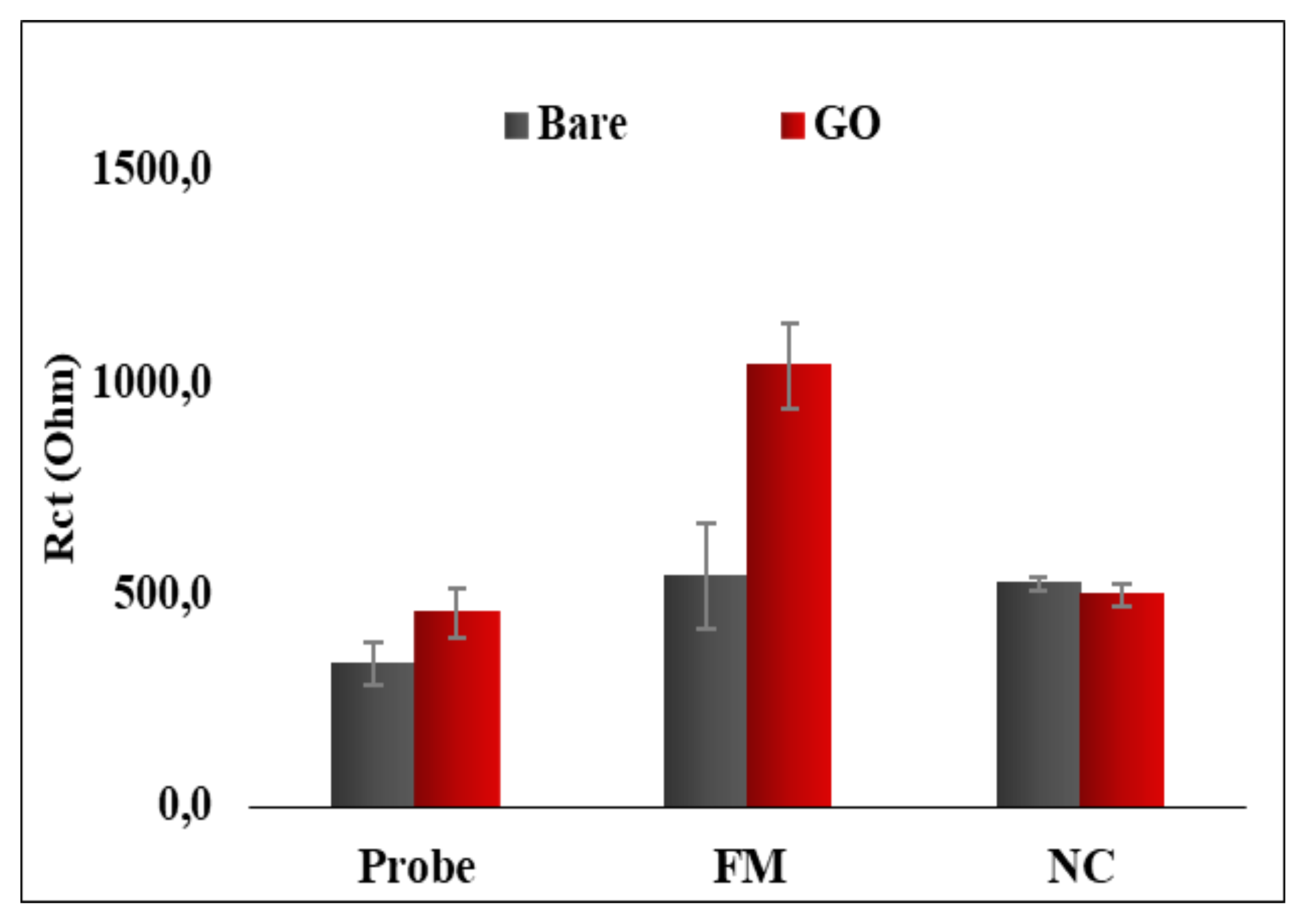

Supplement: Figure S5 — Histogram plot of Rct values provided using; bare PGE and GO-modified PGE under the same conditions: probe (before hybridization), FM (after hybridization with probe and complementary target), NC (after hybridization with probe and non-complementary target). [file tjc-48-05-733s5.tif]

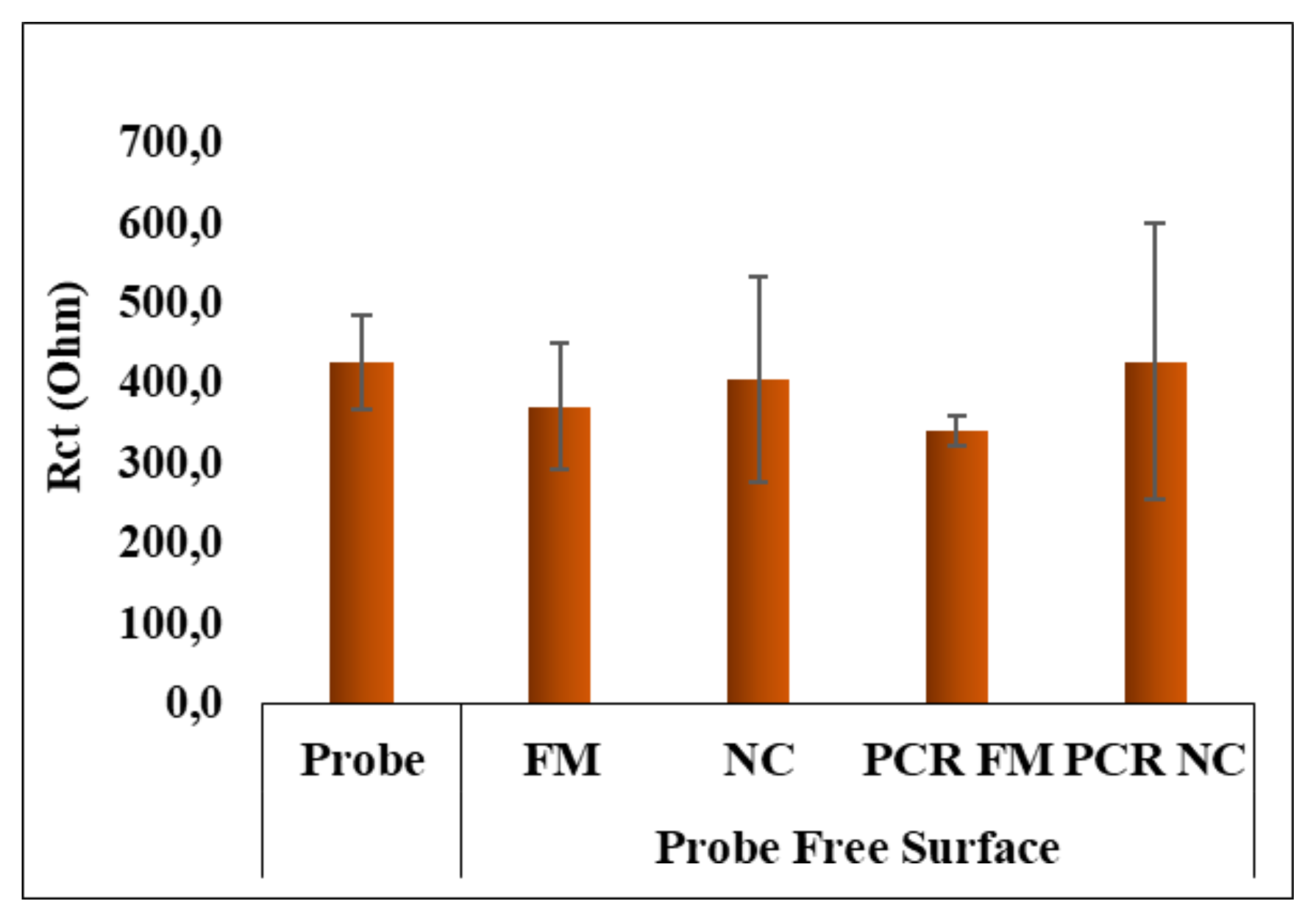

Supplement: Figure S6 — Histogram plot of Rct values provided using; synthetic sequences and PCR amplicons at optimal conditions probe-free surfaces on GO-PGE sensor surfaces. [file tjc-48-05-733s6.tif]
